# Overview of diagnosis and management of paediatric headache. Part I: diagnosis

**DOI:** 10.1007/s10194-011-0297-5

**Published:** 2011-02-27

**Authors:** Aynur Özge, Cristiano Termine, Fabio Antonaci, Sophia Natriashvili, Vincenzo Guidetti, Çiçek Wöber-Bingöl

**Affiliations:** 1 Department of Neurology, Mersin University School of Medicine, Mersin, Turkey; 3 Child Neuropsychiatry Unit, Department of Experimental Medicine, University of Insubria, Varese, Italy; 4 University Center for Adaptive Disorders and Headache (UCADH), Unit of Pavia, Pavia, Italy; 5 Department of Psychiatry of Childhood and Adolescence, Medical University of Vienna, Vienna, Austria; 6 Department of Child and Adolescent Neuropsychiatry, University La Sapienza, Rome, Italy; 7 Department of Psychiatry of Childhood and Adolescence, Medical University of Vienna, Währinger Gürtel 18–20, 1090 Vienna, Austria

**Keywords:** Headache, Childhood, Paediatric headaches, Diagnosis, Epidemiology, Defining features

## Abstract

Headache is the most common somatic complaint in children and adolescents. The evaluation should include detailed history of children and adolescents completed by detailed general and neurological examinations. Moreover, the possible role of psychological factors, life events and excessively stressful lifestyle in influencing recurrent headache need to be checked. The choice of laboratory tests rests on the differential diagnosis suggested by the history, the character and temporal pattern of the headache, and the physical and neurological examinations. Subjects who have any signs or symptoms of focal/progressive neurological disturbances should be investigated by neuroimaging techniques. The electroencephalogram and other neurophysiological examinations are of limited value in the routine evaluation of headaches. In a primary headache disorder, headache itself is the illness and headache is not attributed to any other disorder (e.g. migraine, tension-type headache, cluster headache and other trigeminal autonomic cephalgias). In secondary headache disorders, headache is the symptom of identifiable structural, metabolic or other abnormality. Red flags include the first or worst headache ever in the life, recent headache onset, increasing severity or frequency, occipital location, awakening from sleep because of headache, headache occurring exclusively in the morning associated with severe vomiting and headache associated with straining. Thus, the differential diagnosis between primary and secondary headaches rests mainly on clinical criteria. A thorough evaluation of headache in children and adolescents is necessary to make the correct diagnosis and initiate treatment, bearing in mind that children with headache are more likely to experience psychosocial adversity and to grow up with an excess of both headache and other physical and psychiatric symptoms and this creates an important healthcare problem for their future life.

## Definition

Headache is the most common somatic complaint in children and adolescents both in clinical and epidemiological databases. The incidence of childhood migraine and frequent headache has substantially increased over the last 30 years. The increased incidence is alarming and probably reflects untoward changes in children’s lifestyles. Primary headaches (especially migraine and tension-type headache, TTH) is the most important cause of headaches in this age group, but secondary headaches and unusual causes of headaches also have to be considered [1, 2].

## Epidemiology of headaches

There is a high incidence, prevalence, and individual and societal cost of headache disorders in children and adolescents [3]. The reported prevalence of headache among schoolchildren varies greatly, from 5.9 to 82%, depending on the definition criteria [1, 4–6]. The vast majority of headaches is primary and classified as migraine or TTH.

Prevalence of headache increases throughout childhood reaching a peak at about 11–13 years of age in both sexes [7]. By age 3, headache occurs in 3–8% of children [8, 9]. At age 5, 19.5% have headache and by age 7, 37–51.5% have headache [10–13]. In 7–15-year-olds, headache prevalence ranges from 26 to 82% [12–14].

The studies based on parental reports may be an unreliable source of information on the frequency of headache in young children. It has been suggested that child-completed diaries and teacher observation forms should be used more widely [15]. A population-based study showed that almost 36% of the parents of children with headache are unaware of the headache [16]. Whether ID Migraine TM^®^ is a useful tool in screening adolescent migraine is still under discussion [17].

## Natural history of headache

According to several authors, longitudinal studies and repeated cross-sectional surveys are reported as essential for enhancing the knowledge about the prognostic development of pain disorders and perceived health in the younger population, and for further investigation of possible causal relationships and related factors. Recently, several clinical and epidemiological studies have been published on the long-term course of primary headaches in children and adolescents [18–27].

### Natural history of migraine

Outcome research for paediatric migraine headaches is limited, thus restricting knowledge of the effectiveness of long-term management and outcome. Multidisciplinary treatment was found to be effective for children and adolescents with improvement of multiple outcome variants of paediatric migraine care, including frequency, severity, and school days missed [18].

Some important points could be summarised as follows;

- Diagnoses of primary headache subtypes change over time due to overlapping symptoms and possibly related to maturation.
- Long-term prognosis of headache is adversely affected by an initial diagnosis of migraine and by changing headache location, and it tends to be affected by an increasing time between headache onset and first presentation.
- Girls and children with frequent headache have a poorer prognosis and therefore intervention is particularly important in these groups.
- Stressful life events in childhood have an impact on the course of migraine and TTH and increase the possibility of combined headaches.
- Headache onset early in life increases the risk of an unfavourable clinical course and also genetic factors play an important role in the phenotypic expression of the disease.
- More long-term comprehensive population-based studies are needed in this area.

## How to diagnose headache

A thorough evaluation of headache in children and adolescents is necessary to make the correct diagnosis and initiate treatment. The evaluation should include detailed history of children and adolescents (including parent and teacher observations, observations of child-carer, family relationships, medical history of children and parents) and completed by detailed general and neurological examinations. One has to keep in mind that some symptoms may be referred from the child’s behaviour only (e.g. stopping to watch a favourite movie, interrupting a computer game, or the child’s wish to go to bed in a quiet, darkened room during daytime). Children may also be asked to draw a picture of what their headache, since children, especially younger ones, communicate better through pictures than verbally [28, 29].

### History

The history determines the correct diagnosis, so questions need to be directed to both the child and parents. The following questions should be included:

- Do you have one or more types of headache?
- How did the headaches begin?
- When did the headaches begin?
- Are the headaches progressive, staying the same or improving?
- How often does each headache type occur every month (or every day)?
- How long do the headaches last?
- Do the headaches occur at any special time or under any special circumstances?
- Are the headaches related to specific foods, situations, medications or activities?
- Are there warning symptoms before headache onset?
- Where is the pain located?
- What is the quality of the pain?
- Are there associated symptoms during the headaches?
- What do you do during your headaches?
- What makes the headaches better?
- Does anything make the headaches worse?
- Do symptoms continue between headaches?
- Are you being treated for or do you have any other medical problems?
- Do you take medication for any other problem on a regular on intermittent basis?
- Does anyone else in the family have headaches?
- What do you think is causing your headaches?
- How is your daily routine?

Useful strategies to help improve headache diagnosis in children might be the following:

1. Take the history with sufficient time and patience, and with age-appropriate terminology
2. Ask the patient (assisted by the parents) to keep an appropriate headache diary (e.g. depicting the main headache characteristics and associated symptoms) over a period of some weeks to document the headache frequency and duration, the degree of disability and the occurrence of associated symptoms as well as the use of medications.
3. Give yourself enough time for each patient visit. History should also include pregnancy period of mother, birth history, developmental history, injuries, operations and dietary habits of early childhood, school experiences, history of substance abuse, family relationships, socioeconomic and psychosocial status both of the child and parents.

### Assessment of headache severity

Headache severity of children and adolescents should be quantified using a pain rating scale, visual analogue scale or other equivalents according to age and cognitive levels of subjects. Combined scales may be more useful than one way scales. Biological parameters of pain and observations of other family members should be noted also [30].

### Physical examination

The examiners should keep in mind the tentative diagnosis and substantiate their clinical impression while performing general examination. Important clues should be noted, for example fever may indicate an infection, elevated blood pressure may indicate a hormonal or renal disturbance, growing abnormalities may indicate pituitary or hypothalamic disorders, petechia or palpable lymphadenopathies may indicate haematopoietic abnormalities, organomegaly may indicate a systemic neoplasm, atopic disorders may be related to migraine, and unexplained injuries of different ages may indicate child maltreatment [25, 31, 32].

### Neurological examination

A complete neurological examination should be performed focussing particularly on level of consciousness, meningeal signs, visual disturbances, focal neurological deficits, disorders of coordination, gait and speech, auditory disorders, measurement of head circumstances, localised tenderness of scalp or any body areas. In addition, a psychiatric interview of children and parents should be performed when needed. In the majority of patients with primary headache disorders, the general physical and neurological examinations are normal [4, 33].

### Psychological examination

Repeated pain experiences have some negative effects on daily living activities (i.e. sleep, appetite, play, attention, etc.). During the prepuberty and puberty period changes of emotional status and personality stand in the forefront. It should be differentiating whether the emotional problem or change is a comorbidity or the main problem. Symptoms of depression, which include sadness, tearfulness, withdrawal from activities, hopelessness, need to be checked.

It has been shown that migraine is not related to family and housing conditions, school situation, or peer relations, whereas TTH is associated with a higher rate of divorced parents and fewer peer relations [34]. As an associative comorbidity, the frequency of migraine headache in a clinic sample of Tourette syndrome subjects was nearly fourfold more than the frequency of migraines reported in the general population [35]. The evaluation process should be completed with scales (including depression, anxiety, self-esteem, CBCL, etc.) and family interview.

### Laboratory tests

The choice of laboratory tests rests on the differential diagnosis suggested by the history, the character and temporal pattern of the headache, and the physical and neurological examinations. On the contrary to migraine, detailed laboratory and imaging screen should be performed in case of migraine equivalents [36]. Subjects who have any signs or symptoms of focal/progressive neurological disturbances should be investigated by cranial computed tomography (CT) or magnetic resonance imaging (MRI) [37]. Emergency setting studies showed that neuroimaging (head CT scan or MRI) was performed on 8–41% of children, which on first glance appeared high given that 96% of all patients were ultimately diagnosed with a benign disease. However, 5.5–25% of those who underwent neuroimaging were ultimately diagnosed with a “pathological” process [38–41]. The electroencephalogram (EEG) and neurophysiological examinations (including VEP, event related potentials, EMG, etc.) are of limited value in the routine evaluation of headaches, except from “migraine-triggered seizures” [42, 43]. There are some suggestive clues about pathophysiological association between migraine attacks and epileptic seizures too [44–46]. Lumbar puncture is useful in determining the presence of infection or blood or increased intracranial pressure.

## Primary and secondary headaches

As a general rule IHS classification system divides headache into primary and secondary headache disorders. In a primary headache disorder, headache itself is the illness and headache is not attributed to any other disorder. Primary headaches comprise migraine, tension-type headache, cluster headache, other autonomic cephalgias and other primary headache disorders. In secondary headache disorders, headache is the symptom of identifiable structural, metabolic or other abnormality. In the case of secondary headaches, special attention must be paid to symptoms of increased intracranial pressure and progressive neurological dysfunction. Red flags include the first or worst headache ever in the life, recent headache onset, increasing severity or frequency, occipital location, awakening from sleep because of headache, headache occurring exclusively in the morning associated with severe vomiting and headache associated with straining. Secondary headaches may occur in an acute (such as subarachnoid haemorrhage), subacute (such as meningitis) or progressive (such as neoplasms) fashion.

In children and adolescents, the abrupt onset of severe headache is most frequently caused by upper respiratory tract infection with fever, by sinusitis or by migraine. Serious conditions such as brain tumours or intracranial haemorrhages are uncommon and, when present, are usually accompanied by neurological signs such as papilledema, hemiparesis or ataxia [43].

Both epidemiological and clinical studies have shown that most common causes of headaches in children and adolescents are migraine and TTH.

## Migraine

Migraine is a heterogeneous disorder: attacks vary in pain intensity, duration, pattern of associated features, and frequency of occurrence. Some migraineurs have recurrent attacks without remission periods; others experience symptom-free intervals lasting several years; a third group becomes free of attacks for the rest of their life [47].

Migraine is the second most common cause of chronic recurrent headache in school children. The prevalence ranges from 3.2 to 14.5% [4–6, 26, 47–49]. Positive family history for headache is commonly reported with a frequency of 60–77.5% [4, 22].

Over the last five decades, several definitions of paediatric migraine have been proposed. Vahlquist [50], followed by Bille [1], Prensky and Sommer [51] have been followed by IHS proposing a new set of criteria [52]. Revising the IHS headache duration criterion, i.e. decreasing minimum headache duration from 2 to 1 h, the utility of the IHS criteria for migraine performed 47–86.6% sensitivity and 92.4–98.6% specificity [53–56]. The currently accepted classification system for migraine was published by the International Headache Society in 2004 and is known as the International Classification of Headache Disorders (ICHD-II) [57].

Modification of ICHD-II criteria to include bilateral headache, headache duration of 1–72 h, and nausea and/or vomiting plus two of five other associated symptoms (photophobia, phonophobia, difficulty thinking, light-headedness, or fatigue), in addition to the usual description of moderate to severe pain of a throbbing or pulsating nature worsening or limiting physical activity, improved sensitivity of migraine diagnosis to 84.4% [47, 58].

Balottin [25] demonstrated that the ICHD-II criteria are poorly applicable to children under the age of 6 years. Therefore, the development of alternative criteria might be useful [59, 60]. Further changes in ICHD-II criteria for paediatric migraine could stem from researches comparing the occurrence of headache in the family members and the prevalence of osmophobia in large samples of migraine and TTH patients. Both osmophobia and positive family history could thus become useful in better differentiating migraine and TTH. The prevalence of osmophobia during migraine attacks was 18.5%, and was higher in migraine patients (25.1%) than in those with TTH (8.3%). Osmophobia showed more specificity than phonophobia or photophobia in the differential diagnosis between migraine and TTH [25, 61].

Most migraine symptoms included in ICHD-II are not specific for the paediatric age groups. Among various migraine characteristics and associated disorders only type of migraine, migraine frequency, vomiting and dizziness were related to age [62]. Vomiting may help the diagnosis of migraine in young children with a familial history of migraine and dizziness is more common in children >11 years old and may aid the diagnostic process in this age group [62].

A bidirectional relationship between migraine and depression suggests a neurobiological link. Adverse experiences particularly childhood maltreatment, may alter neurobiological systems, and predispose to a multiplicity of adult chronic disorders. The majority of the studies with clinical populations show slightly higher scores on at least one of the anxiety or depression scales in the migraine group as compared to the control group. However, in all eleven studies, the average score on the anxiety and depression scales obtained by children with migraine did not reach a pathological level, according to the norms established by the validated scales. Findings point to above average levels of anxiety or depression, rather than diagnosed psychopathologies. Therefore, certain authors use the term “sub-clinical”. None of the three studies carried out in the general population revealed differences between the anxiety and depression scores in children with migraine as opposed to children in the control group. The difference in results from studies in the general population and clinical populations can most likely be explained by a recruitment bias. Studies conducted with clinical populations recruit subjects from specialised medical consultations for children and adolescents with migraine, who are probably not representative of the general population. These results contradict those found in the adult population. More studies are needed to better clarify the links between anxiety, depression, and migraine in children, adolescents and adults. The association of childhood sexual abuse with migraine and depression is amplified if abuse also occurs at a later age [20, 34, 63–65].

To ensure the validity of future studies, the following remarks should be taken into account.

- The distinction between headache and migraine is not always clear, even when ICHD criteria are used.
- The children considered to have migraines often have a variety of diagnoses.
- Studies should only use the ICHD second edition criteria.
- Children suffering from migraine are usually recruited from specialised headache centres in hospitals. This is a very specific population and probably not representative of children with migraine in the general population.
- In contrast, studies including patients from specialised centres are relevant too, since they are reflecting the situation in those patients actually seen by physicians.

### Migraine variants

#### Familial hemiplegic migraine (FHM)

FHM is an uncommon and genetically heterogeneous autosomal dominant subtype of migraine with aura in which the aura consists of hemiparesis. Three subtypes of FHM have been described: FHM1, FHM2 and FHM3. Mutations in the genes CACNA1A12 and SCNA1A13, encoding the pore-forming alpha-1 subunits of the neuronal voltage-gated Ca^2+^ channels and Na^+^ channels, are responsible for FHM1 and FHM3, respectively. Mutations in ATP1A2,14 encoding the alpha-2 subunit of the Na^+^, K^+^ ATPase, are responsible for FHM2. The gene mutations for FHM are associated with phenotypes that show an overlap between migraine and other paroxysmal disorders [i.e. CACNA1A and episodic ataxia; SCNA1A and generalised epilepsy with febrile seizures plus (GEFS+)]. These findings provide compelling evidence for ion channels as key targets for preventive migraine treatment [66–69].

#### Basilar-type migraine

Basilar-type migraine is a migraine variant that is classified as part of the spectrum of migraine with aura in the ICHD-II classification. The diagnostic criteria comprise vertigo, visual disturbances in both hemifields, bilateral sensory symptoms and ataxia. The sudden appearance of diplopia, vertigo and vomiting must prompt consideration of disorders within the posterior fossa such as arteriovenous malformations, cavernous angiomas, tumours or congenital malformations [70–72].

#### Ophthalmoplegic migraine

Ophthalmoplegic migraine (OM) is one of the most clinically challenging migraine variants and, fortunately, one of the least common (annual incidence of 0.7 per million). It has been classified by the Headache Classification Committee of the International Headache Society (IHS) in 2004 under the heading of ‘Cranial neuralgias and central causes of facial pain’ [11, 15]. OM is defined as consisting of at least two episodes of headache accompanied or followed within 4 days of its onset by paresis of one or more of the third, fourth and/or sixth cranial nerves, with investigations having ruled out parasellar, orbital fissure and posterior fossa lesions. Contrast-enhanced magnetic resonance imaging performed during symptomatic and postsymptomatic periods in patients with ophthalmoplegic migraine may hold great value in identifying the pathophysiological features of oculomotor nerve palsies. Of cases demonstrating abnormal magnetic resonance imaging, a majority show improved but persistent changes on repeat imaging [73–75].

#### Retinal migraine

Retinal migraine is extremely uncommon in children and usually seen in young adults. Unlike the descending curtain-like onset of amaurosis fugax, retinal migraine causes patients to experience brief (seconds to <60 min), sudden, monocular blackouts or “grayouts” or bright, blind episodes of visual disturbance before, after or during headache attacks [71, 76].

#### “Alice in Wonderland” syndrome

Originated from Lewis Carol’s novel and characterised by bizarre visual illusions and spatial distortions which precede headaches. The children may describe bizarre or vivid visual illusions such as micropsia, macropsia, metamorphopsia and teleopsia [71].

#### Acute confusional migraine (ACM)

This rare type of migraine described as acute confusional states, lasting 4–24 h, associated with agitation and aphasia commonly seen in juvenile migraineurs. ACM may be a presenting feature and important clue, enabling CADASIL to be recognised. Therefore, a brain MRI and/or testing for Notch3 mutations should be considered in adult patients with ACM [77–79].

#### Migraine equivalents

Migraine equivalents of infancy, childhood, and adolescence are recognised periodic, paroxysmal syndromes without associated headache that are thought to be migrainous in aetiology. Following equivalents are presently recognised.

1. Cyclical vomiting (ICHD-II 1.3.1)
2. Abdominal migraine (ICHD-II 1.3.2)
3. Benign paroxysmal vertigo (ICHD-II 1.3.3)
4. Benign paroxysmal torticollis (ICHD-II A1.3.5)

Analgesic overuse may cause a worsening of non-cephalic pain in patients with extra-cephalic variants of migraine [57, 80].

### Diagnosis of migraine

The diagnosis of migraine rests mainly on clinical criteria, thus a correct evaluation begins with a thorough medical history followed by a complete physical and neurological examination including examination of the optic fundus. Recently, a practice parameter that outlined guidelines for the clinical and laboratory evaluation of children and adolescents with recurrent headaches [71] stated that the routine use of any diagnostic studies is not indicated when the clinical history has no associated risk factors and the child’s examination is normal.

## Tension-type headache

Although TTH and migraine are the two most common types of headache in children and adolescents, most articles address migraine headache. The smaller genetic effect on TTH than on migraine suggests that the two disorders are distinct. However, many believe that TTH and migraine represent the same pathophysiological spectrum [81].

### Prevalence

TTH was reported less common in children under 10–12 years of age and more frequent in adolescents, but with reservations for methodological differences and interpretation of results, most of the epidemiological studies found that TTH was the most frequent headache in children aged 8–12 years. The prevalence of TTH in schoolchildren has been reported as 0.9–72.8% relating to study design and psychosocial events. The prevalence of TTH increases with age [5, 13, 81–83].

### Diagnosis of TTH

TTH may be hard to differentiate from migraine in children as some of the symptoms overlap. Regarding the frequency of TTH ICHD-II differentiates infrequent episodic TTH occurring less than once a month, frequent episodic TTH present on up to 14 days per month and chronic TTH occurring at least on 15 days per month or 180 days per year. TTH is characterised by a bilateral pressing tightness occurring bilaterally anywhere on cranium or suboccipital region. The pain is mild to moderate in intensity and usually not aggravated by physical activity. Associated symptoms are absent or limited to one out of photophobia and phonophobia in episodic TTH and one out of mild nausea, photophobia and phonophobia in chronic TTH [57, 81].

### Stressors in TTH

Anxiety and psychological stress factors are often present and headache symptoms may be triggered by additional stressful situations [84].

Underlying psychological stress factors should be evaluated. In children, a connection seems possible between TTH and psychosocial stress, psychiatric disorders, muscular stress, or oromandibular dysfunction. Childhood TTH is associated with a higher rate of divorced parents and fewer peer relations as well as an unhappy family atmosphere. In addition, children with episodic TTH were more likely to report somatic complaints and family problems than those without headache. Children and adolescents with chronic diseases and stressful family events have an increased risk for chronic TTH. Of children with chronic TTH, over 50% have had predisposing physical or emotional stress factors. Compared to migraine group, children with TTH had greater psychological and temperamental difficulties [34, 84–87]. A headache diary is a useful method for the differentiation of headache types. The diagnosis of TTH requires exclusion of secondary headaches.

## Cluster headache and other trigeminal autonomic cephalgias

Cluster headache (CH), the most painful of the primary headaches is a disorder with well-known diagnostic criteria. The condition usually begins in the second decade of life; the prevalence of childhood onset is approximately 0.1% and the sex ratio is in favour of men (M:F ~3.2:1), but with a wide variation of range (1:1–6:1). Onset may be as soon as 3 years, but there is a relatively low number of cases with onset <10 years old. A suspected case in a 1-year-old infant has also been described [88–90]. There are relatively few reports on the prevalence and clinical features in CH in children and adolescents, since only few population studies have also considered the paediatric population [88, 91, 92].

Paroxysmal hemicrania is a rare headache with a prevalence of 0.02%. Paroxysmal hemicrania generally begins in adulthood with onset generally after the third decade of life. Characterised by brief, unilateral attacks of intense pain around the supraorbital and temporal region, afflicted patients may have from usually 5–6 to as many as 30 attacks per day that last from 2 to 45 min. Like other trigeminal autonomic cephalgias, paroxysmal hemicrania is associated with autonomic symptoms. A key element defining paroxysmal hemicranias is their exquisite sensitivity to indomethacin. Relatively few paediatric cases have been reported in the literature. Children as young as 3 years of age have been described with the disorder [93–95].

Short-lasting unilateral neuralgiform headache attacks with conjunctival injection and tearing (SUNCT) is an extremely rare disorder in childhood with only few cases reported in the literature Unlike paroxysmal hemicrania, SUNCT syndrome is unresponsive to indomethacin, and neither oxygen nor other non-steroidal anti-inflammatory drugs provide relief [93, 96].

## Chronic headache

Chronic headache is frequently seen in children and adolescents. ICHD-II provides separate definitions for the chronic forms of migraine, tension-type headache, cluster headache, paroxysmal hemicrania and several secondary headaches. In addition there are primary and secondary headaches which are chronic per se, most importantly medication overuse headache. In ICHD-II, the definition of “chronicity” is heterogeneous. In migraine, TTH or medication overuse headache, it is defined by headaches present on 15 or more days per month for at least 3 months, whereas different chronicity in CH or paroxysmal hemicrania refers to the lack of remission periods [50].

In contrast to ICHD-II, the term chronic daily headache (CDH, with or without differentiating the specific ICHD subtype) is frequently used in the literature. Being aware of this heterogeneity of definitions we did not exclude studies referring to CDH from this review. Chronic headache is estimated to occur in up to 5% of adults and is the most common headache type reported in headache clinics. In children and adolescents, chronic headache is an exceptionally challenging type of headache to treat. The most important subtypes are chronic migraine (CM), chronic tension-type headache (CTTH) and new daily persistent headache (NDPH) [97]. Chronic headache has different expressions in children and adults; the different expressions may reflect several different aetiologies or a developmental continuum. Although a positive family history predisposes children to develop headache, many environmental, biological and psychological processes may share a role in the aetiology [98, 99].

Comorbid chronic migraine and CTTH was the most frequent subtype of CDH (53%). Stressors that precipitated or contributed to the maintenance of CDH were judged important in 63% of the sufferers. Psychiatric disorders are notable in CDH (about 64% of patients) and predict (mainly anxiety) a poorer outcome. Physical abuse (10% vs. 0, *p* = 0.012) and parental divorce (17% vs. 3%, odds ratio = 5.8, *p* = 0.015) were more frequent in the CDH group. The results indicate that childhood adversities may contribute to greater risk of the development of CDH in young adolescents [100–102].

NDPH is the least studied form of CDH. Most adolescents with NDPH do not overuse acute medication and most have prominent migraine features. Therefore, diagnostic criteria should require abrupt onset of a primary CDH of long duration as the sole requirement for NDPH diagnosis [99].

## Other primary headaches

These types of headaches are very rare in childhood and adolescent practice. Some of them are responsive to adequate doses of indomethacin. Before the diagnosis of benign primary headache disorders symptomatic causes (the “crowded” posterior fossa, brain tumours, Chiari malformation, syringobulbia and vascular malformations) should be excluded [103].

## Secondary headaches

Secondary headaches are also called “organic headaches” by some clinicians. These headaches can be grouped in three different ways: aetiology, symptom complex and temporal presentation [104]. Chronic headache in childhood is rarely due to serious intracranial pathology. Some of the important causes of secondary headache disorders are follows.

- Trauma
- Vascular disorders
- Hydrocephalus and neoplasms
- Substance use
- Intracranial infections
- Metabolic disorders and hypoxia
- Disorders of cranium (e.g. sinuses, eyes, etc.).
- Epileptic disorders (both of ictal epileptic headache and differential diagnosis from other benign focal idiopathic epilepsy of infancy).

### Some important clues about secondary headache disorders can be summarised as follows

- Careful history-taking and thorough clinical examination will identify patients with serious underlying brain abnormalities. A change in headache symptomatology or personality should lower the threshold for imaging. However, there is no role for routine neuroimaging in the management of children with primary headache disorders [104–110].
- Headaches occurring soon after trauma frequently involve loss of consciousness, post-traumatic amnesia, or abnormal neurological symptoms and signs, post-traumatic headaches should be kept in mind.
- Minor head trauma could trigger primary headaches (especially migraine) in young children.
- Vascular disorders including vasculitis, hypertension, thrombosis, emboli, and haemorrhage, the latter being secondary to aneurysms, vascular malformations, and trauma are rare, but life-threatening causes of headaches in children and adolescents.
- In progressive headaches associated with signs of increased intracranial pressure, hydrocephalus, idiopathic intracranial hypertension and intracranial hypertension secondary to metabolic, toxic and hormonal causes should be considered.
- Intracranial tumours are the second most common type of neoplasm in children. Symptoms are often unspecific, depending not only on the localization of the tumour, but also on the age of the child. In the majority of patients, the neurological examination will be abnormal, and diagnosis should be confirmed by neuroimaging. It also should be kept in mind that non-neoplastic mass lesions may present in a similar fashion.
- In children presenting with fever, rash, lethargy, irritability, a bulging fontanel, neck stiffness, mental status changes, and/or focal neurological abnormalities intracranial infections should be kept in mind.
- Headaches are seen in patients with medication overuse and use of substances such cocaine, narcotics and amphetamines with or without associated neurological and autonomic symptoms.
- Among headache associated conscious disturbances epileptic disorders should be kept in mind.
- Compound and mixed types of astigmatism, anisometropia and miscorrection of refractive error are found more often in patients with headache than in control subjects.
- Acute sinusitis often presents with fever, rhinorrhea and tenderness over the facial area, as well as headaches. Although the 25% of patient who have been diagnosed as sinusitis previously had at least one sinusitis related complaint, this finding does not seem to be important, because 60% of the patients do not report improvement after sinusitis treatment.
- Misdiagnosis of primary headache disorders should be kept in mind.

## Conclusions

- Headache in children and adolescent is a growing problem possibly related to changing lifestyle and stressors.
- Families and physicians need more knowledge about headaches in children and adolescents.
- Headache diagnosis may be more difficult in these age groups due to declaration problems and overlapping symptoms.
- In each visit of a subject with a primary headache disorders a secondary cause of headache should be kept in mind.
- Headache evaluation should be including cognitive functions and impact on daily living activities.
- Comorbidities must be considered.
- Headache diary is a mandatory tool for diagnosis and effective follow-up in patients with recurrent headaches.
- Children with headache are more likely to experience psychosocial adversity and to grow up with an excess of both headache and other physical and psychiatric symptoms and this creates an important healthcare problem for their future life.

Taking careful history from a patient presenting with headache is the prerequisite for further diagnostic and therapeutic management.
